# Clinical and radiological features of idiopathic interstitial pneumonias (IIPs): a pictorial review

**DOI:** 10.1007/s13244-014-0335-3

**Published:** 2014-05-22

**Authors:** Stefano Palmucci, Federica Roccasalva, Silvia Puglisi, Sebastiano Emanuele Torrisi, Virginia Vindigni, Letizia Antonella Mauro, Giovanni Carlo Ettorre, Marina Piccoli, Carlo Vancheri

**Affiliations:** 1 Radiodiagnostic and Radiotherapy Unit, University Hospital Policlinico-Vittorio Emanuele, Via Santa Sofia 78, 95123 Catania, Italy; 2 Regional Centre for Interstitial and Rare Lung Diseases, Department of Clinical and Molecular Biomedicine, University Hospital Policlinico-Vittorio Emanuele, Via Santa Sofia 78, 95123 Catania, Italy

**Keywords:** Radiology, Idiopathic interstitial pneumonias, Classification, Lung disease interstitial, Pulmonary Fibrosis, Idiopathic Pulmonary Fibrosis

## Abstract

**Objectives:**

To illustrate the clinical and radiological features of idiopathic interstitial pneumonias (IIPs), according to the American Thoracic Society (ATS)/European Respiratory Society (ERS) classification updated in 2013.

**Methods:**

IIPs include a subset of diffuse and restrictive lung diseases, resulting from damage to the parenchyma characterised by inflammation and fibrosis of the interstitium. Classification into major and rare IIPs is based on the 2013 ATS/ERS committee.

**Results:**

The diagnosis of idiopathic pulmonary fibrosis (IPF) needs to exclude other well-known causes of interstitial lung diseases. According to the 2011 evidence-based guidelines, usual interstitial pneumonia (UIP) can be diagnosed by HRCT when all criteria are fulfilled. Non-specific interstitial pneumonia (NSIP) is characterised by patchy ground-glass opacities and irregular linear/reticular opacities. The imaging patterns of respiratory bronchiolitis associated-interstitial lung disease (RB-ILD) and desquamative interstitial pneumonia (DIP) show centrolobular nodules and ground-glass opacities. Cryptogenic organising pneumonia (COP) consists of patchy peripheral or peribronchial consolidations, while ground-glass opacities are typically associated with diffuse lung consolidation, evolving to fibrosis, in acute interstitial pneumonia (AIP). Rare IIPs include lymphoid interstitial pneumonia and idiopathic pleuro-parenchymal fibroelastosis (IPPFE).

**Conclusions:**

The knowledge of IIP imaging features on HRCT images help radiologists in diagnosis. Moreover, the overlap of imaging features needs a multidisciplinary approach.

***Teaching Points*:**

• *UIP findings are reticulations, bronchiectasis, honeycombing and absence of inconsistent features.*

• *Bilateral patchy ground-glass areas represent the most encountered features in NSIP.*

• *Poorly defined centrilobular nodules are typical of RB-ILD, whereas a ground-glass appearance is typical of DIP.*

• *HRCT features of COP include characteristic peripheral or peribronchial patchy consolidations.*

• *Rare IIPs include idiopathic LIP and idiopathic pleuro-parenchymal fibroelastosis (PPFE).*

## Introduction

The idiopathic interstitial pneumonias (IIPs) are a group of acute and chronic diffuse parenchymal lung diseases with many important features in common. Although all IIPs are characterised by an unknown aetiology [1, 2], a strong correlation with many factors has already been well established in the literature [3, 4].

In 2002 The American Thoracic Society (ATS) and the European Respiratory Society (ERS) published a classification of IIPs [5]. This classification includes usual interstitial pneumonia (UIP), non-specific interstitial pneumonia (NSIP), desquamative interstitial pneumonia (DIP), respiratory bronchiolitis-associated interstitial lung disease (RB-ILD), cryptogenic organising pneumonia (COP), acute interstitial pneumonia (AIP) and lymphoid interstitial pneumonia (LIP).

The ATS/ERS Classification has been recently updated, distinguishing major IIPs from rare IIPs (Table 1). Major IIPs are grouped into “chronic fibrosing” (IPF and NSIP), “smoking related” (RB-ILD and DIP) and “acute/subacute” (COP and AIP). Rare IIPs include idiopathic LIP and idiopathic pleuro-parenchymal fibroelastosis (PPFE) [6].

**Table 1:** IIPs classification and their HRCT features

IIPs			HRCT features
*Major*	*Chronic fibrosing IIPs*	*UIP*	*-Reticular pattern, with or without transaction bronchiectasis*
			*-Honeycombing apperance*
			*-Basal and subpleural predominance*
			*-In “UIP pattern” absence of features listed as inconsistent with UIP*
		*NSIP*	*-Bilateral ground-glass areas*
			*-Reticular opacities*
	*Smoking-related IIPs*	*RB-ILD*	*-Poorly defined centrilobular nodules*
			*-Centrilobular emphysema and/or bronchial wall thickening*
		*DIP*	*-Diffuse ground-glass opacities*
			*-Irregular linear opacities*
			*-Microcyst*
	*Accute/subacute IIPs*	*COP*	*Peripheral or peribronchial patchy consolidations*
			*-Ground-glass opacities with a tendency to migration*
			*Rarely mass or nodules that may cavitate (“atoll sign”)*
		*AIP*	*-Ground-glass attenuation areas with a mosaic pattern*
			*-Air space consolidation in dependent area*
*Rare*	*LIP*		*-Perivascular cysts and ground-glass opacities*
			*-Centrilobular and subpleuralnodules*
	*PPFE*		*-Subpleural thickening of apital regions*
			*-Small subpleural consolidations*

IIPs are generally distinguished from other diffuse parenchymal lung diseases on the basis of clinical properties, laboratory tests, physical examination, imaging and histopathological features [1, 2, 7, 8]. Nevertheless, in some cases it is very hard to distinguish the condition from other diseases such as asbestosis, chronic hypersensitivity pneumonitis, autoimmune disorders and sarcoidosis [9, 10]. In addition, IIP's diagnosis should exclude other diffuse lung diseases such as emphysema or chronic obstructive lung disease, diffuse lung disease associated with occupational or environmental exposure, lymphangioleiomyomatosis, histiocytosis and eosinophilic pneumonia [8].

The role of the radiologist in the diagnosis of IIP has being continuously emphasised in recent years because of the significant contribution of HRCT in the identification and diagnosis of diseases. In fact, according to the 2011 evidence-based guidelines, UIP can be diagnosed by HRCT when all criteria are fulfilled [11].

This pictorial review focusses on the main clinical and radiological features of IIPs in order to help residents, general radiologists and clinicians in their diagnosis. However, there is an overlap of imaging features among IIPs, and a multidisciplinary approach is strongly recommended.

## Methods

### Chronic fibrosing IIPs: UIP and NSIP

#### Usual interstitial pneumonia (UIP)

Idiopathic pulmonary fibrosis (IPF) is defined as a “specific form of chronic, progressive, fibrosing interstitial pneumonia of unknown cause, occurring in adult lungs and associated with the histopathological and/or radiological pattern of UIP” [11]. Incidence and prevalence are not easy to estimate because there were different definitions of IPF [11].

The annual incidence of IPF in the USA is estimated at 6.8–8.8 per 100,000 [12], while the annual incidence is between 0.22 and 7.4 per 100,000 in the UK [13]. In the USA Coultas reported that males have a higher prevalence of IPF than females [14]; Raghu reported a higher IPF prevalence among older males (>65 years), and a similar trend has been observed in European studies too [12]. A higher prevalence is observed in patients >75 years of age [12, 15].

Comparative epidemiological data of different interstitial lung diseases (ILDs) are limited to a few studies, which suggest that sarcoidosis and IPF are the most frequent ILDs followed by hypersensitivity pneumonitis and ILD in collagen vascular disease [16].

For a long time IPF has been considered the result of a chronic inflammatory process that evolves in fibrosis. Currently, a hypothesis based on the presence of an inflammatory process has been has been abandoned and the “epithelial-fibroblastic route” proposed instead to explain disease development [17]. It consists of a pathological interaction between the pulmonary epithelium and mesenchyme. An initial “noxa patogena” causes injury to alveolar epithelial cells, with their apoptosis. The release of growth factors and chemokines leads to the migration of mesenchymal cells throughout the damaged basement membrane, creating fibroblastic foci (activated fibroblast-myofibroblast). Myofibroblasts secrete extracellular matrix proteins, providing a provisional matrix that serves as the scaffold for normal tissue repair. To reach a correct healing formation, myofibroblasts should undergo apoptosis. Failure of apoptosis is typical of IPF, leading to myofibroblast accumulation, exuberant extracellular matrix production and pathological scar formation. A recent hypothesis proposes IPF as a neoproliferative disorder of the lung. Genetic alterations, response to growth and inhibitory signals, resistance to apoptosis, altered cellular communications and intracellular signalling pathways are hallmarks common to IPF and cancer [18].

Histologically the disease is characterised by scattered fibroblastic foci with heterogeneous distribution that alternate interstitial inflammation and honeycombing. These findings coexist with normal lung areas. The prognosis of UIP is variable. In fact, the disease is influenced by the extent of the disease and the severity of functional impairment at the time of diagnosis [11].

#### Radiological features

**Fig. 1:**
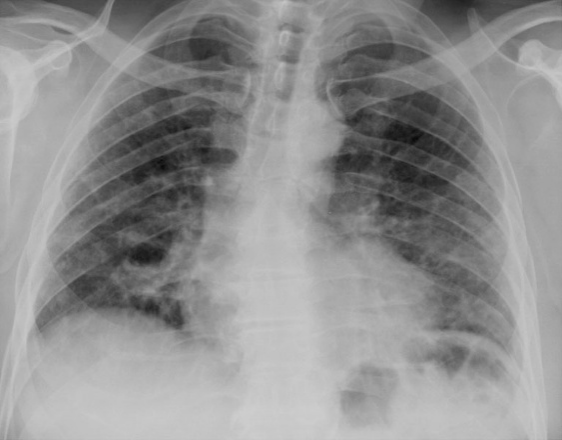
Chest X-ray in a 57-year-old man with UIP. Imaging findings are not specific and reticular pattern in the early stage of disease is not appreciable

Imaging findings on a chest X-ray are not specific (Fig. 1). Reticular pattern and bronchiectasis, involving predominantly lower lobes and costophrenic angles, are generally recognised. Unfortunately, imaging findings could be misdiagnosed in the early stage of disease.

**Fig. 2:**
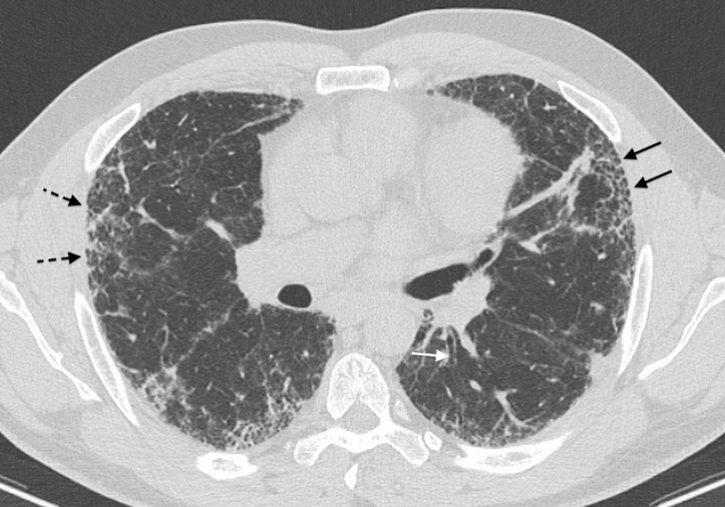
HRCT image of a UIP pattern (same patient of Fig. 1). Typical imaging features of UIP are clearly recognisable because of the presence of honeycombing (black arrows), traction bronchiectasis (white arrow) and a reticular pattern (black dotted arrows). Lesions are typically located along subpleural regions

**Fig. 3:**
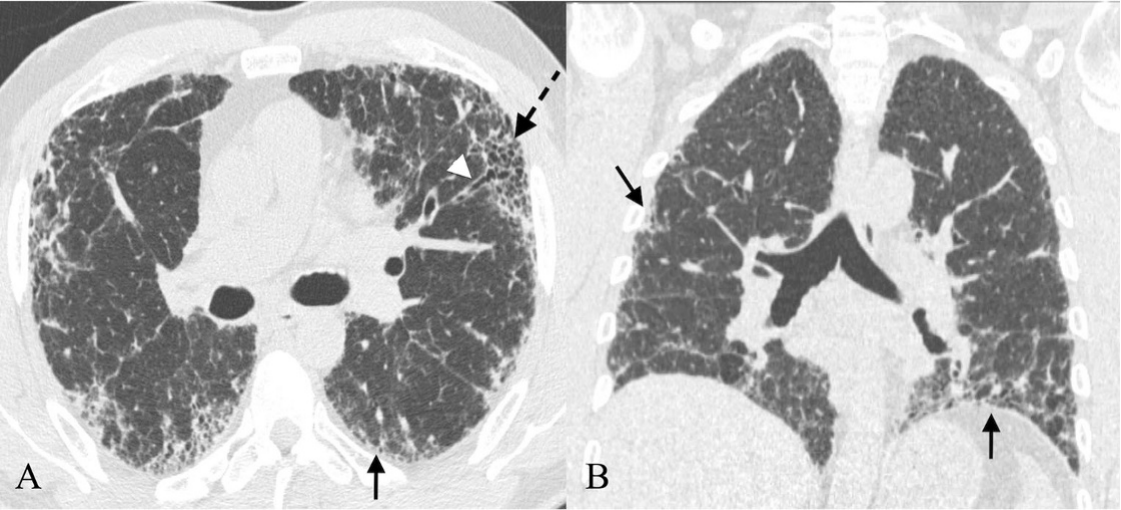
“UIP pattern”. A UIP pattern is characterised by all the following features: (1) basal and subpleural predominance (black arrows); (2) reticular pattern (black arrows), with associated traction bronchiectasis (white arrowhead); (3) honeycombing appearance (black dotted arrow); (4) absence of features listed as inconsistent with the UIP pattern

Typical HRCT features are represented by a reticular pattern, traction bronchiectasis and honeycombing appearance (Figs. 2 and 3). These imaging findings are predominantly located in the subpleural regions and in lower lobes.

**Fig. 4:**
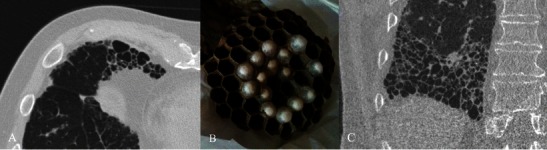
Honeycombing appearance on HRCT images. The cystic spaces reproduced on HRCT images (A and C); a real honeycomb (B, removed from apartment window of one of the authors). The honeycombing appearance consists of a cluster of cystic air spaces, usually with a diameter of 3–10 mm, although they may reach 2.5 cm in size [19, 20]

**Fig. 5:**
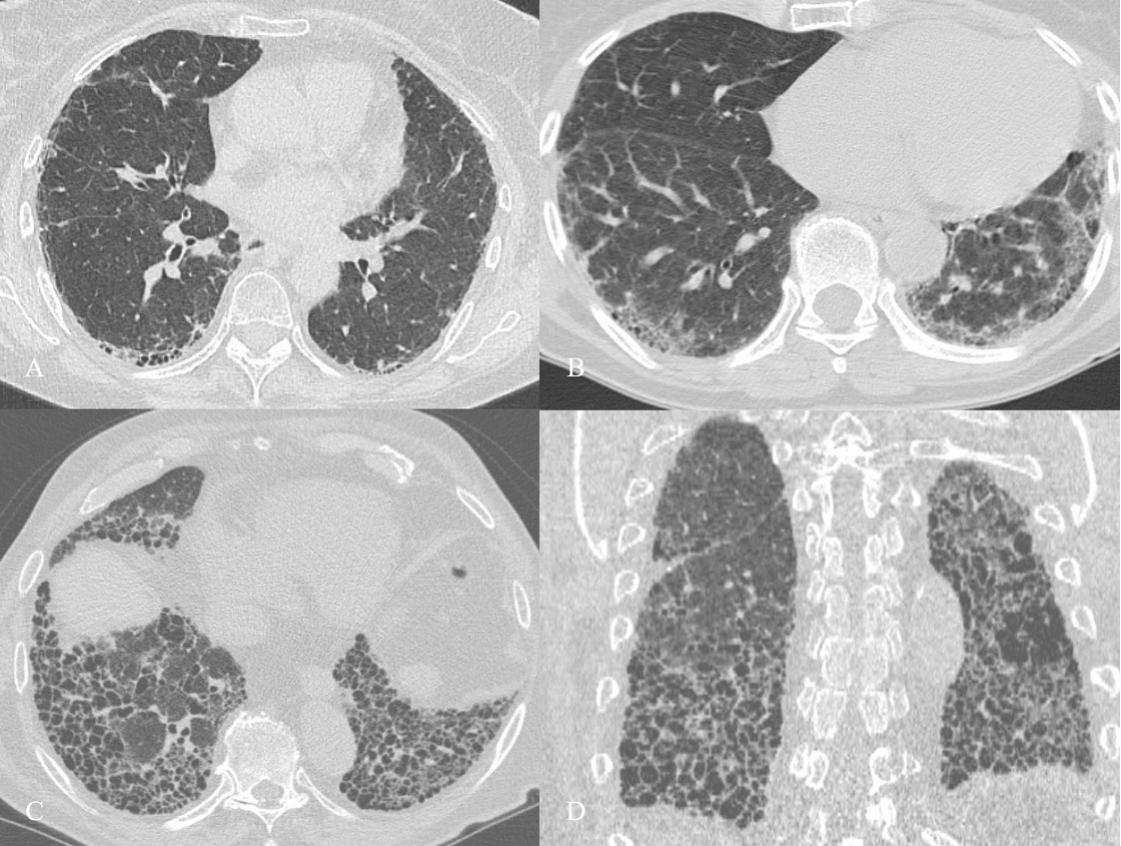
Variable degrees of honeycombing appearance. HRCT images in three different UIP patients, with variable degrees of honeycombing representation. Image A shows a subpleural reticular pattern and small honeycombing area. The diagnosis of UIP was also successfully confirmed by histological biopsy. Patient DLCO was 89 % at the time of diagnosis. In a second patient (B), with respiratory insufficiency and severe reduction of DLCO (39 %), the UIP pattern was again diagnosed by CT examination. C and D show diffuse honeycombing, which massively involves the basal regions of lower lobes. The patient in C and D, at the time of clinical examination, was a smoker of 40 pack/years, with progressive dyspnoea and productive cough for 2 years. Pulmonary function tests showed lung failure and severe DLCO reduction (30 %) with obstructive spirometric pattern

On HRCT, the most important imaging feature is the honeycombing, which consists of a cluster of cystic air spaces, usually with a diameter of 3–10 mm, although they may reach 2.5 cm in size (Figs. 4 and 5) [19, 20]. The honeycombing is considered the most specific sign of UIP [19]. HRCT shows enlargement of mediastinal lymph nodes in 70–80 % of cases. The nodes are typically encountered in the paratracheal areas [10].

Traction bronchiectasis is frequently encountered among IIPs, very often reproducing a micro- or macrocystic appearance that is difficult to differentiate from honeycombing. The use of multiplanar reconstructions (MPR) is strongly recommended for distinguishing between honeycombing and a cluster of cystic bronchiectasis.

The diagnosis of UIP is essentially based on HRCT and histological appearance [11].

Three main patterns have been distinguished: “UIP pattern”, “possible UIP” and “inconsistent with UIP. The UIP pattern (Fig. 3) is characterised by all four features of the disease, which include: (1) basal and subpleural predominance; (2) reticular pattern, with associated traction bronchiectasis; (3) honeycombing appearance; (4) absence of features listed as inconsistent with a UIP pattern [11].

**Fig. 6:**
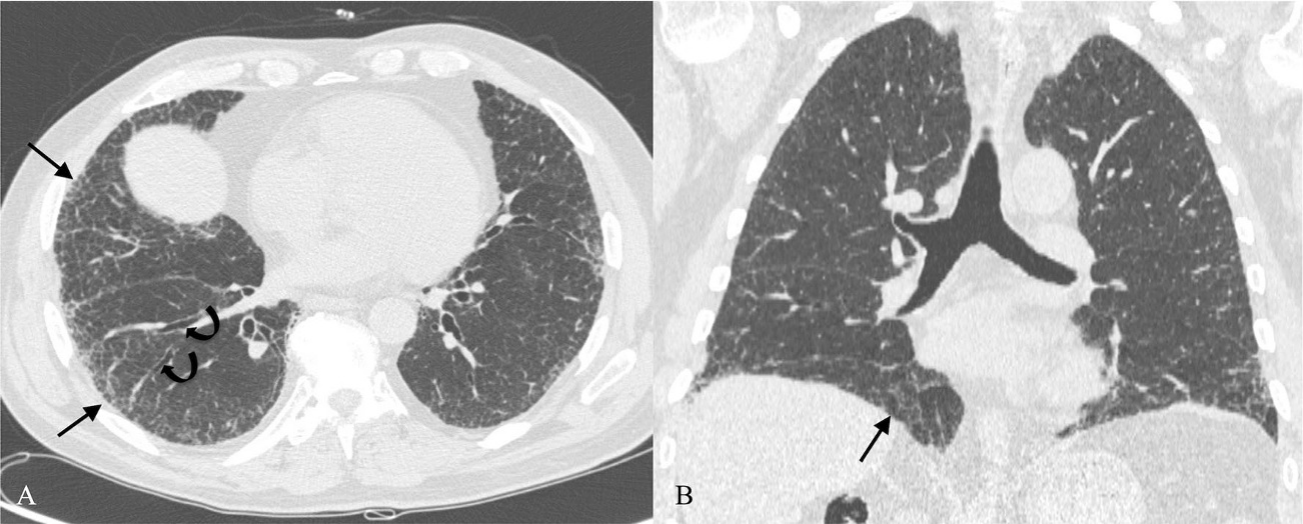
“Possible UIP” pattern in a 77-year-old man. The possible UIP pattern includes: (1) reticular pattern (black arrows), with or without bronchiectasis (curved black arrows); (2) the presence of a typical subpleural location (black arrows); (3) no features listed as inconsistent with the UIP pattern

The possible UIP pattern (Fig. 6) includes: (1) reticular pattern again—with or without traction bronchiectasis; (2) the presence of a typical gradient (lower lobes and subpleural localisation of disease); (3) no features listed as inconsistent with a UIP pattern. The most specific sign of UIP, the honeycombing appearance, is not recognisable.

**Fig. 7:**
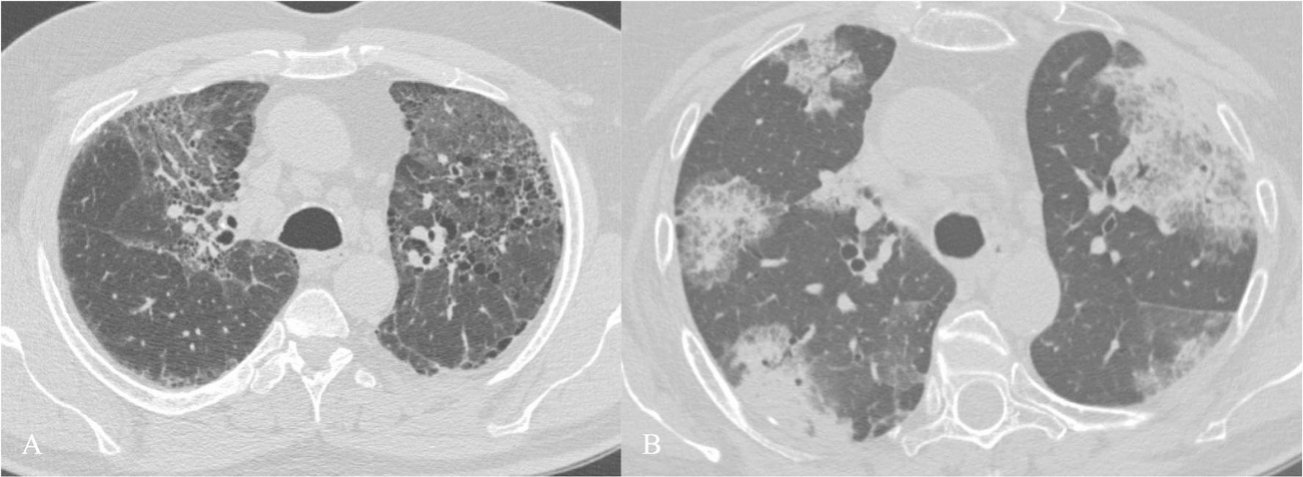
Pattern “inconsistent with UIP”. “Inconsistent with UIP” patterns in two HRCT images respectively found in a 75-year-old woman (A) and a 57-year-old man (B). Cystic spaces along peribronchovascular bundles (A) and parenchymal consolidations (B) are well demonstrated. However, UIP diagnosis was formulated in the histological reports in both cases

Finally, the pattern “inconsistent with UIP” (Fig. 7) includes the presence of any of the following features: upper or mid-lung predominance; peribronchiovascular predominance; extensive ground-glass abnormality; profuse micronodules; discrete cysts; diffuse mosaic attenuation/air-trapping; parenchymal consolidations.

These three patterns need to be related with histological appearances of IPF, even though the role of HRCT is a crucial element in the diagnosis. Matching the three patterns reported with histological results, we have to underline the following innovations:

1. The UIP pattern does not need to be confirmed by histology. HRCT after the exclusion of identifiable causes of ILD allows for the diagnosis of IPF, even if surgical lung biopsy is possible, probable or non-classifiable [11].
2. The possible UIP pattern needs to be correlated with biopsy. Cases with a possible UIP pattern on HRCT and UIP or probable histological UIP pattern allow for the diagnosis of IPF;

The diagnosis of IPF is graded as “possible” when we identify one of the features listed as inconsistent with UIP and surgical lung biopsy obtains a pattern of UIP [11].

#### Non-specific interstitial pneumonia (NSIP)

The introduction of NSIP among IIPs was made by Katzenstein and Myers in 1998. Several studies have been focussed on the fact that some cases of interstitial pneumonias did not match with UIP, DIP or AIP on the basis of the histopathological features [21]. These pneumonias have been reported as “non-specific interstitial pneumonias” by Travis et al. [21, 22].

NSIP is less common than UIP, but is still one of the most common interstitial pneumonias. Its incidence is unknown, with different reports ranging from 14 to 36 % of all IIPs [5, 23].

The histopathological and radiological pattern of NSIP is reproduced in a wide variety of lung diseases, and this is the reason why some authors in the literature have debated considering this disease an independent clinical entity [24]. However, the revision 2002 ATS/ERS IIPs statement classifies idiopathic NSIP as a specific clinicopathological entity [6].

“The clinical diagnosis of NSIP”—as reported by Romagnoli et al.—“should be reserved only for idiopathic and biopsy-proven cases when no causative factor can be identified” [25]. NSIP has been divided into either idiopathic NSIP [5]—when the disease is not related to another underlying disease—or secondary NSIP, which occurs in cases where it is possible to identify a known aetiology [26]. Several articles in the literature describe the associations between the radiological appearance of NSIP and autoimmune disorders [27, 28].

Histologically, NSIP is characterised by temporally uniform interstitial changes in the lung, whereas UIP is typically heterogeneous in the temporal distribution of fibrosis [29]. NSIP, as reported in a paper by Schaefer and Prokop—“shows a temporary more uniform histological picture in the way that parenchymal changes appear to have occurred over a single relatively narrow time span” [10]. Histologically, considering the variable amounts of fibrosis and inflammatory components, Travis et al. distinguished a “cellular NSIP” and a “fibrosing NSIP”. Here, the cellular type is characterised by chronic interstitial inflammation with poor fibrosis, while the fibrosing NSIP shows preservation of the alveolar architecture and interstitial thickening due to fibrosis [30].

The distinction between cellular and fibrosing NSIP has a prognostic implication. Travis et al. revised more than 100 biopsies including UIP, cellular NSIP, fibrosing NSIP and DIP. The survival rate at 5 and 10 years for patients with histologically proven cellular NSIP, fibrosing NSIP and DIP was higher than in patients with idiopathic UIP [22]. In addition, analysing the 5- and 10-year survival rates, they discovered a significant difference between cellular and fibrosis types of NSIP, with a better course observed for cellular NSIP [22].

Mainstream treatment is based on corticosteroids. Among patients treated with corticosteroids—when reduction of the dose is not able to avoid collateral effects—a possible resource could be an additional nonsteroidal immunosuppressive agent.

#### Radiological features

Considering the different prognosis between UIP and NSIP, radiologists need to carefully evaluate the most important imaging features in the differentiation between UIP and NSIP.

Imaging findings on a chest X-ray are not specific, with infiltrates predominantly located in the lower lobes, a reticular pattern and bronchiectasis.

**Fig. 8:**
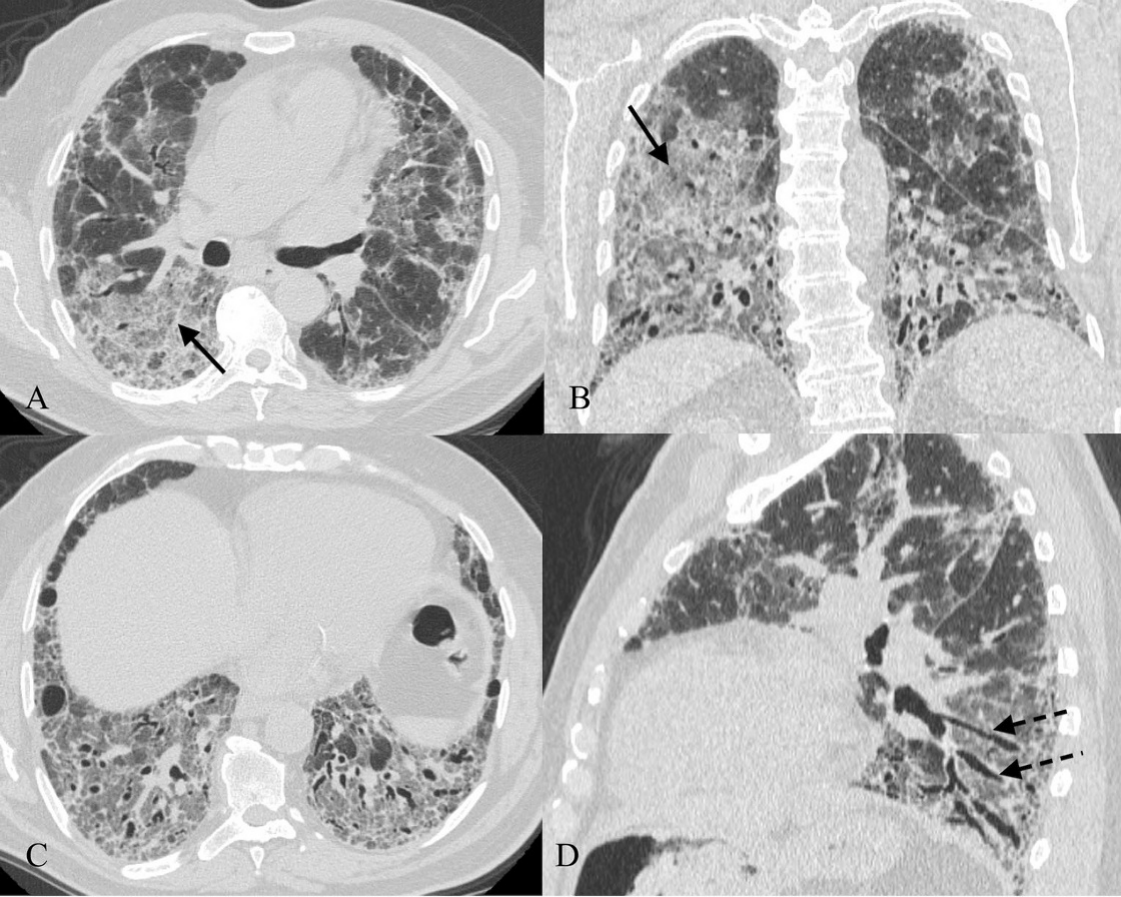
NSIP pattern in a 64-year-old man. HRCT features of NSIP include extensive ground-glass areas in the lung (black arrows) and traction bronchiectasis. This bronchiectasis frequently shows a parallel course through the lung, well depicted by sagittal reconstruction in D (black dotted arrows). There is no honeycombing in the lung. Cystic bronchiectasis is generally well documented by MPR images

**Fig. 9:**
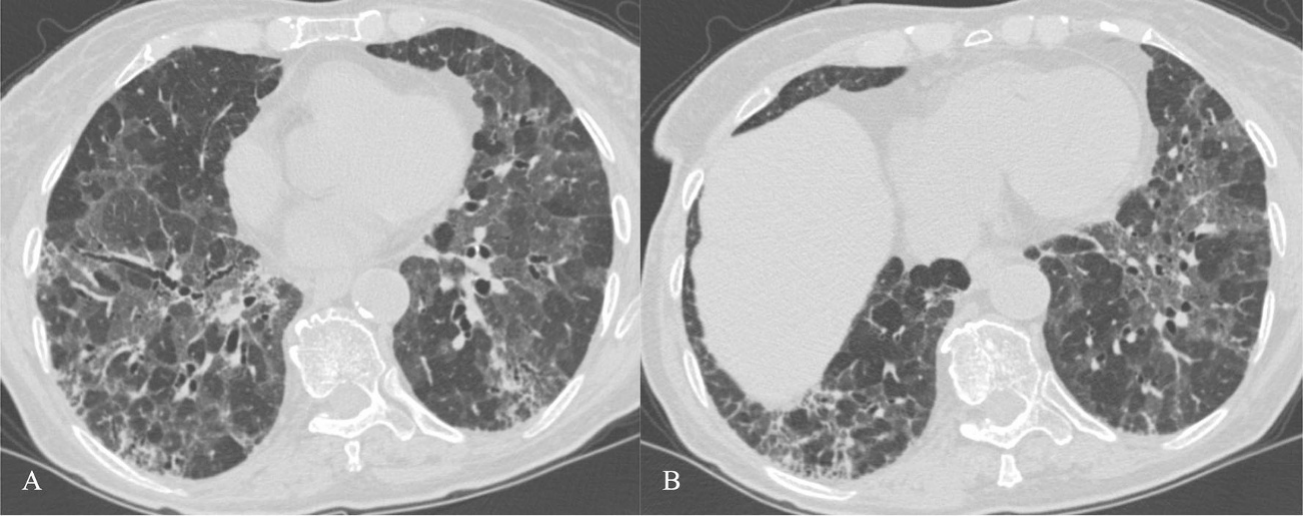
NSIP pattern. Typical NSIP pattern in a 78-year-old woman, consisting of ground-glass areas, sometimes superimposed on a reticular pattern, and traction bronchiectasis

HRCT features (Figs. 8 and 9) include [7, 31, 32]:

- bilateral ground-glass areas in the lung, sometimes with extensive distribution;
- reticular opacities, which can be superimposed on a ground-glass pattern;
- traction bronchiectases, which can be parallel in their course through the lungs or extremely irregular.

**Fig. 10:**
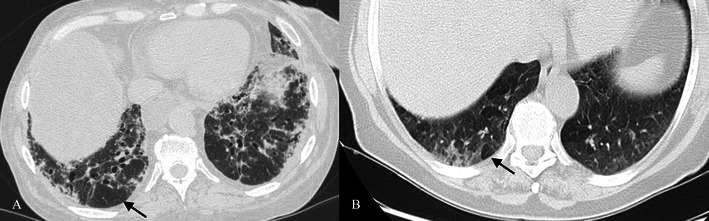
HRCT features of NSIP. NSIP patterns are shown in a 52-year-old woman (A) and in a 75-year-old woman (B). Bilateral patchy ground-glass areas, predominantly located in the middle and lower areas, represent the most important HRCT feature of NSIP. However, when the ground-glass area is not extensively distributed through the lungs, the differential diagnosis from the UIP pattern remains very difficult. Subpleural areas not involved by ground-glass opacifications (black arrows) have been suggested as other CT features helpful for diagnosis [30]

Bilateral patchy areas of ground glass, predominately located in the middle and lower lung, represent the most encountered features in NSIP, with or without traction bronchiectasis or reticulation. Honeycombing is a rare sign among NSIPs [33–35]. Lower lobe volume loss and subpleural areas not involved by ground-glass opacifications (Fig. 10) are other CT features observed [30].

However, in a series of 50 patients studied by Hartmann, the typical pattern of NSIP described—bilateral patchy ground-glass areas—was found only in 22 % of cases; 32 % of patients showed pulmonary alterations compatible with a UIP pattern, and in the remainder of cases the imaging appearance was considered non-diagnostic or more compatible with other chronic infiltrative lung diseases [33, 36]. This variability of imaging presentation correlates with the heterogeneous histological appearance of NSIP. Some authors have previously reported that some cases of NSIP are reported as a “diagnosis of exclusion” in patients who have histological findings not typical of AIP, UIP or COP. These patients have variable degrees of interstitial inflammation and fibrosis, focal areas of COP, prominent inter-alveolar macrophages and peribronchial accentuation of infiltrates [36]. This heterogeneous appearance—with many features of COP, UIP and DIP—explain the variability on imaging [36].

A honeycomb-like appearance could be simulated by irregular traction bronchiectasis. Use of coronal reformatted images helps to easily distinguish between the ectatic cystic-like changes of the bronchi and the true cysts of typical honeycombing [37]. Differential diagnoses include several chronic infiltrative diseases, such as extrinsic allergic alveolitis, UIP, DIP and AIP.

### Smoking-related IIPs: RB-ILD and DIP

#### Respiratory bronchiolitis with associated interstitial lung disease (RB-ILD)

RB-ILD has been defined as the “clinical manifestation of interstitial lung disease associated with the pathological lesion of RB” [5]. The first description of smoking-related bronchiolitis was reported in 1974, when Niewoehner demonstrated the presence of clusters of pigmented macrophages in respiratory bronchioles and neighbouring alveoli of smokers [38]. Myers et al. observed similar alterations—respiratory bronchiolitis—in patients with clinical and radiological features of chronic interstitial lung diseases [39]. The clinicopathological syndrome of RB-ILD was subsequently introduced by Youseum et al. [40].

The histological reports described by Niewoehner—pigmented macrophages in respiratory bronchiolitis [38]—represent a specific response to smoking. In some patients, bronchiole inflammation and intra-alveolar accumulations of pigmented macrophages cause the development of clinical and radiological ILD.

Fraig et al. demonstrated the presence of RB in all smokers and about 50 % of former smokers [41]. It generally affects smokers of 30–40 years of age with a history of more than 30 pack-years of cigarette smoking [42, 43]. The disease has a slight male predominance [44]. The most encountered clinical symptoms are cough and dyspnoea. Inspiratory crackles are found in about 50 % of cases, whereas digital clubbing is rare [45].

The histological hallmark of RB-ILD is represented by the accumulation of alveolar macrophages within respiratory bronchioles. Macrophages are characterised by eosinophilic cytoplasm, with brown granular pigmentation representing constituents of cigarette smoke. It is also possible to observe a chronic inflammatory cell infiltrate within bronchiolar walls, while no honeycomb change or fibroblastic foci are present. Myers and colleagues suggested that the main difference between RB and RB-ILD is based on the extent of the fibrosing and inflammatory process that in RB-ILD involves also adjacent alveolar walls [39].

A number of studies that have linked smoking with the insurgence of RB-ILD have demonstrated a clear improvement of the disease after smoking cessation. Nakanishi et al. have shown that smoking cessation alone, without any other treatment, leads to clinical, functional and radiographic improvement [46]. Symptoms and DLCO values were both improved after smoking cessation as well as ground-glass opacities and centrilobular nodules on CT scans. However, some studies have not confirmed the effectiveness of smoking cessation and have also investigated the role of other treatments such as steroids or immunosuppressant drugs; Portnoy et al. described a clinical and functional decline in 32 patients with RB-ILD, in spite of smoking cessation and steroid treatment [47]. However, the real effectiveness of smoking cessation, corticosteroids or immunosuppressants in the treatment of RB-ILD remains a clinical dilemma and needs to be further evaluated.

#### Radiological features

**Fig. 11:**
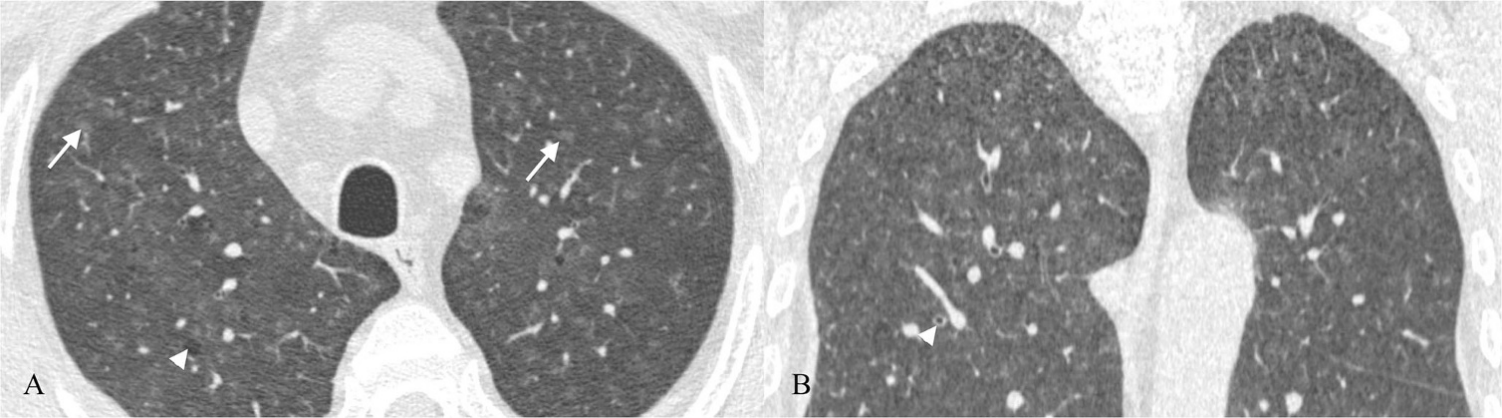
RB-ILD pattern in a 52-year-old smoker patient. Poorly defined centrilobular nodules (white arrows), due to peribronchial extension of the infiltrate, are uniformly disseminated through the upper lobes. Other CT features very frequently encountered are centrilobular emphysema (white arrowhead)

Normal chest X-ray has been described in up to 20–30 % of cases [48]. Non-specific signs often detected are represented by thickening of the central and peripheral wall of bronchi and ground-glass areas [42, 49]. Other possible pattern feature on chest X-ray could be the presence of reticular-nodular opacities with upper lobe predominance or diffuse distribution. Main HRCT features (Fig. 11) are [50]:

- ground-glass opacities (correlated with macrophage accumulation in alveolar spaces);
- poorly defined centrilobular nodules (due to peribronchial extension of intraluminal infiltrate), also indicated as centrilobular ground-glass nodules (Fig. 11).
- diffuse lung distribution;
- other main CT features: centrilobular emphysema and/or bronchial wall thickening due to cigarette smoking and frequently encountered in RB-ILD patients (Fig. 11) [7, 8, 42].

A small number of patients show a reticular pattern due to fibrosis in the absence of traction bronchiectasis and honeycombing [43, 44, 49]. Differential diagnosis includes acute hypersensitivity pneumonitis, NSIP and DIP [44].

#### Desquamative interstitial pneumonia (DIP)

The term “desquamative interstitial pneumonia” was introduced by Liebow, who believed that intra-alveolar cells, typical of this disease, were reactive alveolar pneumocytes “desquamated” from the alveolar surface [51]. Later, electron microscopy demonstrated that these cells were alveolar macrophages, even though the definition of “desquamative interstitial pneumonia” is currently considered a form of interstitial pneumonia characterised by the presence of diffuse exudation of pigmented macrophages in the alveolar spaces [6]. Some studies have proposed DIP as the cellular phase of UIP because of some similarities in histopathological features, but this idea was not sustained by Carrington et al. because of the poor prognosis for UIP and the absence of response to corticosteroid therapy in comparison with DIP [52].

The correlation between cigarette smoke and DIP has been proved in different studies, showing that almost 90 % of patients affected by DIP are current or former smokers. Based on a comprehensive evaluation of HRCT findings, Heyneman et al. hypothesised that DIP and RB-ILD may be considered as different degrees of severity of a reaction of small airways and lung parenchyma to cigarette smoke [49]. It is important to note that DIP has also been associated with a variety of other conditions including drug reactions and connective tissue diseases such as scleroderma [53], lupus [54] and rheumatoid arthritis [55]. Very few epidemiological data regarding DIP are present in the literature, probably linked to the poor knowledge of the disease. According to Carrington et al., DIP accounts for less than 3 % of interstitial lung diseases, commonly affecting patients in their third to fifth decade, with a preference for males, who are affected nearly twice as often as females [52].

Even though the criteria to differentiate histological patterns of DIP from RB-ILD are well defined [6], the histopathological diagnosis of DIP is difficult because of the similarities to RB-ILD. According to this classification, DIP is characterised by macrophage accumulation in the distal air spaces with alveolar pneumocyte proliferation along the alveolar septa. The thickening of alveolar septa is also due to a chronic inflammatory infiltrate that includes eosinophils and lymphoid aggregates. However, the histological differential diagnosis between DIP and RB-ILD is based on the extension of lesions. DIP affects the lung in a more uniform manner, while RBILD lesions have a bronchiolocentric distribution [49].

#### Radiological features

Chest radiographs may show bilateral interstitial infiltrates (hazy opacities) but this pattern is non-specific for detection of DIP.

**Fig. 12:**
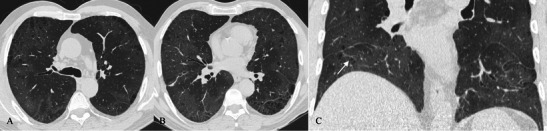
DIP pattern in a 60-year-old man. The patient (smoker of 25 pack-years) complained of progressive dyspnoea and dry cough. Pulmonary function was characterised by a moderate reduction in DLCO and normal dynamic lung volumes. Physical examination revealed digital clubbing and fine crackles were heard in the lung bases on chest auscultation. On HRCT images, lung ground-glass opacifications are clearly shown through the lungs and correlate with the intra-alveolar accumulation of macrophages. Bronchial wall thickening and bronchiectasis are also associated. Microcysts have been described in almost 50 % of cases [7]. The coronal HRCT image (C) clearly shows peribronchovascular small cysts (white arrow); also emphysema is recognisable through the lung

**Fig. 13:**
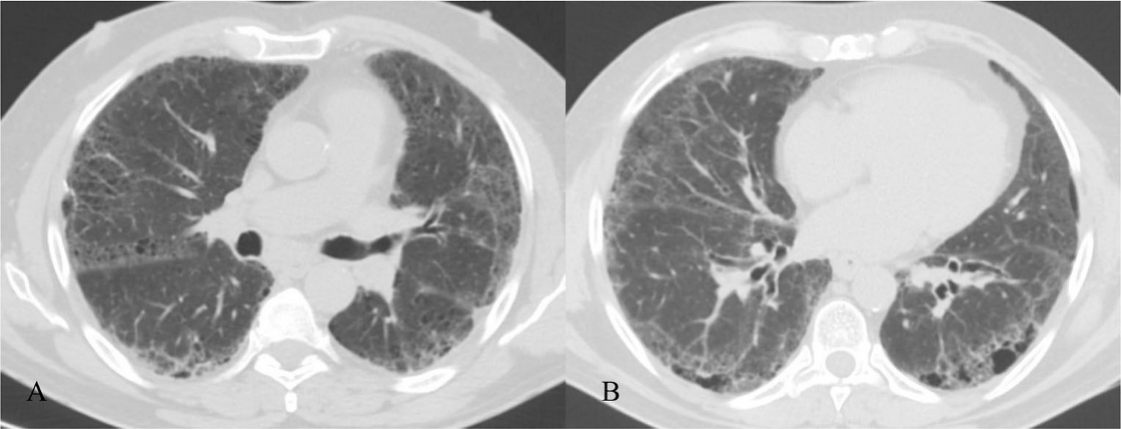
DIP pattern. A 54-year-old male smoker with DIP (diagnosis confirmed at histological examination) showing ground-glass opacifications and cysts

Pathognomonic radiological features of DIP are well studied through HRCT scans (Figs. 12 and 13). They are represented by:

- Diffuse ground-glass opacities (Figs. 12 and 13), correlated histologically with the spatially homogeneous intra-alveolar accumulation of macrophages and thickening of alveolar septa: bilateral and symmetric (86 %), basal and peripheral (60 %), patchy (20 %) and diffuse (20 %) [8, 56, 57].
- Irregular linear opacities.
- Microcysts (50 % of patients) [7] (Fig. 12).

However, ground-glass opacification of the lung is the most important imaging feature of DIP. Differential diagnosis generally includes chronic hypersensitivity pneumonia and RB-ILD.

### Acute/subacute IIPs: COP and AIP

#### Cryptogenic organising pneumonia (COP)

Descriptions of this clinical entity first appeared in the latter half of the nineteenth century, notably in a lecture by J.M. Charcot in Paris in 1877–1878 [58]. Milne described a pneumonia “where the usual process of resolution has failed and organisation of the inflammatory exudate in the air alveoli of the lung by fibrous tissue has resulted” [59]. COP is a well-established entity known since the 1950s. However, at the beginning, it was considered a pulmonary condition caused by chronic persistence of a pneumonitis.

COP has also been called idiopathic bronchiolitis obliterans organising pneumonia (BOOP). This term was described for the first time by Davidson in 1983, but was changed to avoid confusion with constrictive bronchiolitis obliterans, which is an airway disease [60]. Actually, the term COP is preferred because its clinical, physiological and imaging features are unrelated to bronchiolar obliteration [1].

COP still is included in the classification of IIPs published by Travis et al. [6] in view of its idiopathic nature. Sometimes it is possible to identify an underlying disease, so the sentence “OP associated to” is generally suggested [6].

The mean age of patients affected by COP is 55 years. There is no gender predilection. COP is not associated with cigarette smoking. In fact, most patients are nonsmokers or ex-smokers. Histopathologically it is characterised by the accumulation of granulation tissue lying within small airways, alveolar ducts and alveoli [61]. In particular, the intra-alveolar granulation tissue consists of intermixed myofibroblasts, fibroblasts and connective matrix [62]. The intra-alveolar buds in COP may resemble some histopathological features of fibroblastic foci of usual interstitial pneumonia, but differently the myofibroblasts do not show progression to irreversible fibrosis [62]. The origin of these myofibroblastic cells has already been debated in several articles. Some recent studies have shown that myofibroblasts are not resident in the pulmonary interstitium, but they derive from bone marrow [63].

The main clinical features include subacute onset of cough, fever, dyspnoea and sparse crackles at auscultation [64].

#### Radiological features

Imaging findings of COP have already been described; it can be observed in three main patterns: the typical form, due to the presence of multiple alveolar opacities; the form with focal consolidation, also called focal COP; the infiltrative form, characterised by the presence of infiltrative opacities.

On a chest X-ray, the typical appearance consists of multiple bilateral patchy areas of consolidations [61]. These consolidations are due to the presence of granulation tissue at the alveolar spaces. Typically, these alveolar infiltrates could migrate to different lung areas on different chest X-rays.

**Fig. 14:**
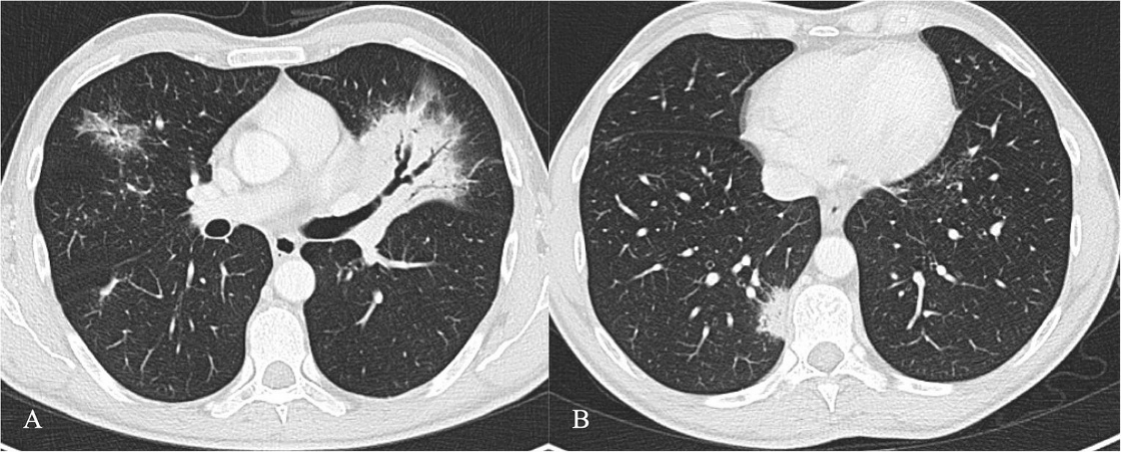
COP pattern in a 53-year-old man. HRCT images show characteristic peripheral and bronchocentric consolidations. The “air bronchogram sign” is clearly recognisable in A. Tendency to migration and changing location are also typical imaging findings of COP
- increase of consolidations over several weeks after antibiotic therapy;
- ground-glass opacities with tendency to migration, change of location and size;
- rarely a mass or nodule that may cavitate or reproduce the typical appearance of an “atoll sign” (Fig. 15);

HRCT features of COP are more extensive than chest radiographs and include:

- characteristic peripheral or peribronchial patchy consolidations (sometimes with subpleural area spread), also with air bronchograms and mild cylindrical bronchial dilatation (Fig. 14);

**Fig. 15:**
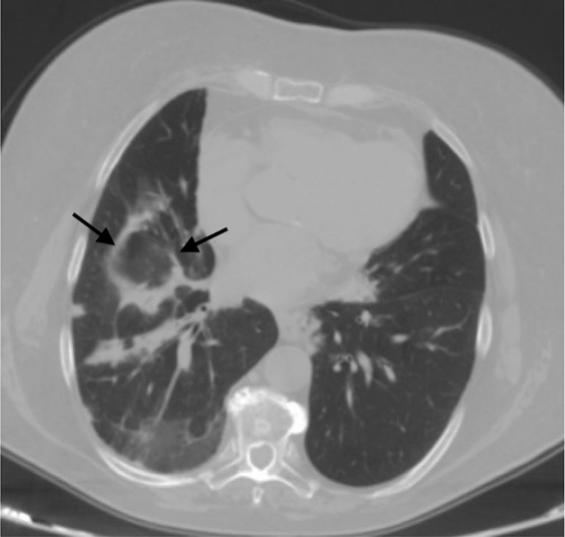
An “atoll sign” in a 69-year-old female. Black arrows show circular consolidations surrounding normal parenchyma

The “atoll sign” is due to the presence of a ring consolidation surrounding normal lung or ground-glass opacification. It is considered relatively specific for OP as it is found in 20 % of patients with COP. However, the atoll sign has also recently been described in other conditions such as sarcoidosis and Wegener’s granulomatosis [65–67]. HRCT shows multiple consolidations or nodules distributed in sub-pleural areas or along the bronchovascular bundles [61, 68, 69].

#### Acute interstitial Pneumonia (AIP)

AIP is severe and sudden in onset, progressing rapidly [70]. The disease has been reported by Hamman and Rich, describing for the first time “four cases of a rapidly progressive disease pathologically characterised as extensive pulmonary fibrosis” [71, 72]. Katzeinstein reported other cases very similar to those observed by Hamman and Rich and in 1986 introduced the term AIP for these “rapid interstial lung diseases” [73].

AIP represents an idiopathic disease, which occurs more or less equally in men and women, with a mean age of 50 years [7, 74, 75]. It usually occurs in previously healthy people, although often the patients present with symptoms suggestive of a viral upper respiratory infection, such as myalgia, arthralgia and fever. Smoking might not increase the risk of developing AIP.

Most patients develop severe dyspnoea within 3 weeks of the onset of symptoms and seek medical attention with signs of pneumonic consolidation with diffuse crackles [5, 7, 74]. A restrictive pattern with reduced diffusing capacity is typical in pulmonary function tests. The condition rapidly progresses to acute respiratory failure, which requires mechanical ventilation, with oxygen supplementation.

Diagnostic clinical criteria for acute respiratory distress syndrome (ARDS)—consisting of acute onset, a PaO2/FIO2 (fraction of inspired oxygen ratio) equal to or less than 200 mmHg, diffuse bilateral opacities on chest radiograph, and a pulmonary capillary wedge pressure of less than 18 mmHg or no clinical evidence of left atrial hypertension—are often fulfilled [73, 75, 76].

In the early phase of disease, corticosteroid therapy seems to be useful. The prognosis of AIP is poor and death occurs in 50 % or more of cases 1–2 month after illness onset; the patients who survive the acute phase of the disease later develop lung fibrosis [74].

The histological hallmark of AIP is diffuse alveolar damage with the same appearance of acute respiratory distress syndrome caused by sepsis and shock. In fact, AIP is considered an idiopathic form of ARDS [72, 77]. The alveolar damage can be classified into an early exudative phase and a late organising phase, based on the timing between lung insult and biopsy [6, 7].

AIP in the exudative phase is characterised by interstitial and intra-alveolar oedema, hyaline membranes and diffuse alveolar infiltration by acute inflammatory cells. In the organising phase, generally at the end of the first week after lung injury, granulation tissue, with alveolar wall thickening, organising fibrin and fibrosis within alveolar lumens and thrombi in small and medium-sized pulmonary arterioles, are visible [7, 72, 78].

Sometimes, the lung architecture may be partially restored, while in other cases, the lungs develop extensive honeycomb fibrosis, which is uniform with numerous fibroblasts and little collagen deposition, different from the heterogeneous appearance of UIP.

The histopathological pattern is necessary for a definitive diagnosis [7]. Sometimes, ill patients are unable to tolerate surgical lung biopsy and a transbronchial biopsy is performed [7, 79].

#### Radiological features

Chest radiography in AIP shows bilateral airspace opacifications with air bronchograms, which usually spare the costophrenic angles [80]. The lung volume may be lower than normal. Consolidations often become more diffuse and in progression to the organising stage the radiograph shows a ground-glass appearance with irregular linear opacities.

**Fig. 16:**
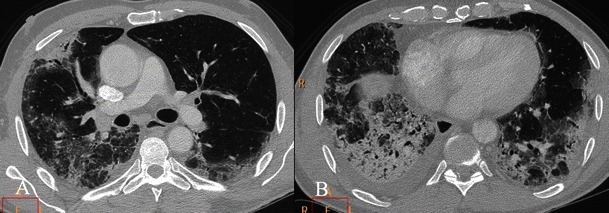
AIP pattern in a 56-year-old man. The patient was admitted to our emergency room for severe dyspnoea (leukocytes = 12,000/mmc; neutrophils 90 %; D-dimer 452 ng/ml; CRP 7,10 mg/dl; ERS 78 mm/h). CT images show mild pleural effusion, ground-glass opacifications and inhomogeneous consolidations. Differential diagnosis includes several diseases, such as acute exacerbations of IIPs, NSIP, COP, infectious diseases and collagen-related lung fibrosis

On HRCT images (Fig. 16), AIP is characterised by diffuse ground-glass attenuation areas with a mosaic pattern, resulting from the presence of alveolar septal oedema and hyaline membranes, and airspace consolidation in dependent areas of the lung, reflecting the intra-alveolar oedema and haemorrhage; these features are seen in the exudative phase [7, 74, 80, 81]. These signs are usually bibasilar, but often can be diffuse or involve upper lobes [74]. Consolidations, caused by intra-alveolar fibrosis, are seen in the organising phase, characterised by lung architectural distortion, traction bronchiectasis and cysts [74, 80–83]. These findings are more severe in the nondependent areas of the lung, because of the “protective” effect of atelectasis and consolidation on the dependent areas of the lung in the exudative phase to attenuate the potential injury related to mechanical ventilation [7, 74, 84]. Patients who survive show areas of hypoattenuation, lung cysts, reticular abnormality and associated parenchymal distortion often in the nondependent lung.

Other differential diagnoses are widespread infection such as *Pneumocystis carinii* pneumonia, DIP, hydrostatic oedema, haemorrhage, alveolar proteinosis and bronchioloalveolar cell carcinoma.

Ground-glass attenuation at HRCT usually evidences the presence of potentially reversible disease. Several studies have shown that in AIP areas of ground-glass attenuation and consolidation are visible in all histological phases, reflecting different histological findings, so they are not helpful in predicting the underlying histology [77, 81, 85]. The association of ground-glass attenuation, consolidation and traction bronchiectasis correlates with the fibrotic phase [77]. Histopathological examination is essential and diagnosis should be made after the revision of all clinico-radiological-pathological data.

### Rare IIPs: idiopathic LIP and idiopathic pleuroparenchymal fibroelastosis

#### Lymphoid interstitial pneumonia (LIP)

LIP was first described by Liebow and Carrington in 1969 in relationship with diffuse lymphocytic infiltrates in the interstitium, different from other patterns of interstitial pneumonia [86–88]. At the beginning it was included in the main classification of IIPs edited by Liebow, but many years later it was removed. Some authors in fact assert that it should be classified among pulmonary lympho-proliferative diseases and not with interstitial pneumonias, because it appears in patients with lymphoma [87, 89–91]. It was also considered a preneoplastic condition, progressing to malignant lymphoma in up to 30 % of cases [74, 87].

In recent years both ATS and ERS have once more classified LIP among IIPs, because it often remains of unknown origin [5, 87].

LIP is rare and it is often associated with systemic disease such as Sjögren's syndrome (25 % in patients with LIP), AIDS (particularly children), immunodeficiency syndromes (Castleman's disease) and autoimmune thyroid disease; these disorders should be investigated when this pattern is histologically found [1, 74, 87, 91]. When other underlying diseases are definitively excluded, a diagnosis of idiopathic LIP could be defined [5].

LIP usually affects women and it is typically diagnosed during the fifth decade of life [87]. Onset of the symptoms is often slow, with patients complaining of a gradually progressive cough and dyspnoea over 3 or more years. Sometimes, systemic symptoms, such as fever, weight loss, chest pain, night sweats and arthralgia, are also reported [87]. LIP rarely progresses to fibrosis, so crackles, digital clubbing and physiological features of other IIPs are unusual [87, 88]. This pneumonia often shows symptoms of the underlying systemic or autoimmune disease, such us lymphadenopathy in the presence of Sjögren’s syndrome. Respiratory symptoms help to detect lung involvement.

Laboratory investigations can detect mild anaemia and dysproteinaemia as a polyclonal increase in gamma globulin or a monoclonal increase in IgG or IgM (approximately in 80 % of patients with LIP). The risk of a lymphoproliferative malignancy increases in patients with a monoclonal gammopathy or hypogammaglobulinaemia [74, 87].

Bronchoalveolar lavage fluid shows numerous lymphocytes, without any clonality at immunophenotyping.

Histologically, LIP seems to be a histological variant of diffuse pulmonary lymphoid hyperplasia primarily affecting the interstitium. It is characterised by lymphoid infiltration of the interstitium with lymphocytes, plasma cells and histiocytes, which lead to progressive widening of alveolar septa [74, 87, 94–96]. The airspace may be secondarily affected by interstitial infiltrates, proteinaceous fluid and macrophage accumulation. Reactive lymphoid follicles are often present along inflamed peribronchiolar areas; nonnecrotising granulomas and honeycombing in advanced cases may also be seen [74]. LIP must be differentiated from follicular brochiolitis, a peribronchiolar lymphocytic infiltrate with germinal centres.

The treatment of LIP consists of corticosteroids. Therapy response is uncertain as many patients present an arrest or improvement in symptoms and more than one-third progress to diffuse interstitial fibrosis. Death often occurs because of infections associated with immunosuppression, progressive pulmonary fibrosis, or transformation to malignant lymphoma.

#### Radiological features

HRCT images usually demonstrate diffuse or predominantly lower bilateral abnormalities. They also show the presence of lymphatic nodes, normally seen in patients with LIP [87]. LIP is most severe in the inflamed perilymphatic interstitium, so thickening of the bronchovascular bundles and interlobular septa is usual [94]. Uniform or patchy areas of bilateral ground-glass opacity are typical findings in LIP and they reflect the histological evidence of diffuse interstitial inflammation [92, 95].

**Fig. 17:**
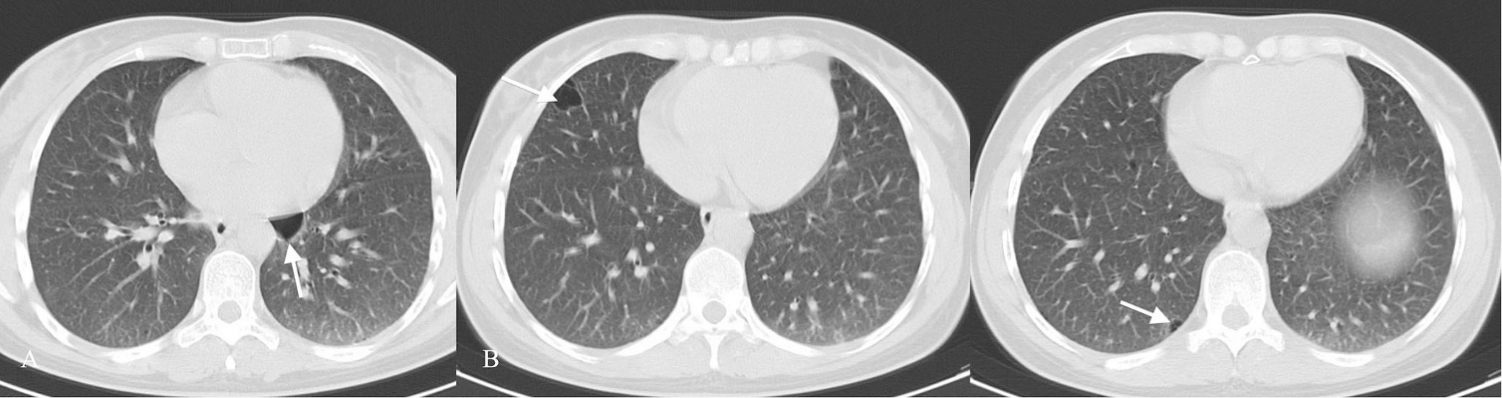
Pattern of LIP. Small thin-walled cysts (white arrows) represent a typical imaging feature of LIP, usually not exceeding 30 mm and located in the mid-lung zones, adjacent to blood vessels. A partial “check-valve” bronchiolar obstruction has been considered the main explanation for cyst formation. The bronchiolar obstruction—due to lymphoid nodules and aggregates—leads to “air trapping”

Other findings (about 80 % of cases) are a few, perivascular, thin-walled cysts (Fig. 17) [74, 94]. These cysts measure 1–30 mm and are seen within the lung parenchyma, predominantly in the mid-lung zones, adjacent to blood vessels (in UIP, cysts are usually subpleural and in lower lung areas) [97, 98]. A partial “check-valve” bronchiolar obstruction has been considered as the main explanation for cyst formation because the bronchiolar obstruction due to the lymphatic infiltration leads to a consequent partial air trapping [97, 98].

The association of perivascular cysts and ground-glass opacities is highly suggestive of LIP.

**Fig. 18:**
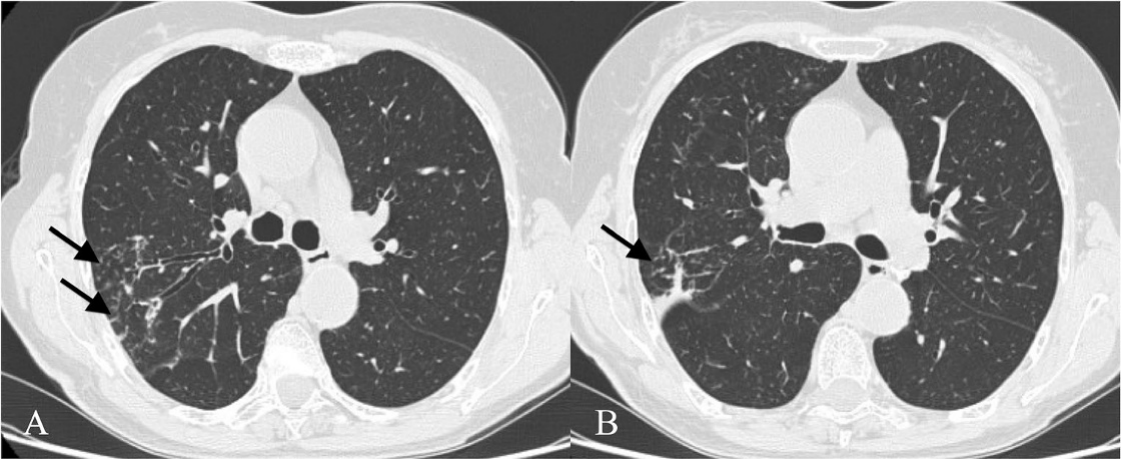
Pattern of LIP (after corticosteroid treatment). Small reticular opacities and nodules (black arrows) are well depicted in the caudal area of the right upper lobe. Surgical biopsy revealed reactive lymphoid follicles along peribronchial regions and indicated a LIP diagnosis

Centrilobular and subpleural nodules (Fig. 18) with thickening of the interlobular septa are also typical and reflect the inflammatory infiltration of the peribronchiolar interstitium [92]. Perivascular honeycombing in areas of previous airspace abnormality and reticular pattern (in about 50 % of patients) can also be seen.

Regarding the differential diagnosis, the HRCT pattern of LIP should be differentiated from hypersensitivity pneumonitis, sarcoidosis and lymphangitic spread of tumour. *Pneumocystis carinii* pneumonia may be considered because of cystic abnormalities. A very important differential diagnosis is from Hodgkin’s lymphoma [99].

#### Pleuroparenchymal fibroelastosis (PPFE)

Idiopathic PPFE has been recently included in the ATS/ERS IIPs classification [6] as a rare IIP. However, it was described by Amidani et al. in the Japanese literature as idiopathic pulmonary upper lobe fibrosis [100, 101].

It is a characterised by the development of fibrosis along pleural surfaces and subpleral parenchymal lung, with predominantly upper lobe involvement.

Some clinical data report an association between recurrent pulmonary infection and development of PPFE. Many authors have recently reported PPFE as a pulmonary reaction associated with chronic graft-versus-host disease following bone marrow transplantation [102]. Some histopathological patterns of PPFE have been interpreted as little spontaneous pneumothoraces followed by an obliterative pleural reaction and subpleural fibrosis [102].

In a PPFE series reported by Reddy et al. a male/female ratio of 1/1.4 was found, whereas in the literature the tendency is a male/female ratio of 2/1 [101].

Histologically, the hallmarks consist of pleural thickening and subpleural fibrosis.

#### Radiological features

Chest X-ray shows aspecific pleural thickening of apical regions, with small subpleural pulmonary consolidations. This imaging pattern is indistinguishable from many other chronic pulmonary infections (tuberculosis). Superior hilar retraction has been described in all five cases reported by Kusagaya et al. [103].

**Fig. 19:**
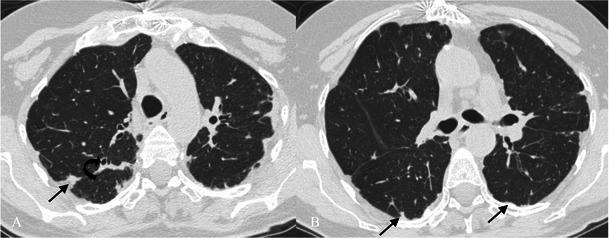
IPPFE pattern in a 57-year-old woman. Pleural thickening and subpleural consolidations (black arrows) are well recognisable in the upper lobes. Sometimes pleural thickening involves the lobar fissure (black arrow in A). Bronchiectases are also encountered (black curved arrow)

HRCT (Fig. 19) shows pleural thickening in the upper pulmonary zones, sometimes also associated with other signs of fibrosis, such as bronchiectasis. Subpleural consolidations and/or reticulations are well depicted in the upper and middle zones. Pleural irregularities could extend to the interlobar fissures [102]. Chronic fibrosis leads to pulmonary volume loss. Other pulmonary IIPs could also be observed in the lung, particularly in the PPFE spared areas [101].

## Conclusions

IIPs diagnosis is currently based on a multidisciplinary approach, and HRCT offers a special contribution because some patterns have been associated with specific IIPs. Consequently, radiologists should be more able to detect ILDs on HRCT images.

According to the 2011 evidence-based guidelines [11], UIP can be diagnosed by HRCT when all criteria are fulfilled. Early recognition of its pattern represents a diagnostic challenge for radiologists, in order to suggest correct disease management (based on recent medical treatment such as pirfenidone).

The overlap of imaging features among interstitial pneumonia limits the diagnosis. NSIP's diagnosis can be difficult because of the heterogeneity of radiological appearances. Smoking-related IIP patients could improve their course with smoking cessation. Clinical presentation could help radiologists in their approach to acute/subacute IIDs (COP and AIP).

## References

[1] Lynch DA, Travis WD, Muller NL, Galvin JR, Hansell DM, Grenier PA, King TE (2005). Radiology. Idiopathic interstitial pneumonias: CT features.

[2] Dixon S, Benamore R (2010). Clin Radiol. The idiopathic interstitial pneumonias: understanding key radiological reatures.

[3] Maher TM, Wells AU, Laurent GJ (2007). Eur Respir J. Idiopathic pulmonary fibrosis: multiple causes and multiple mechanisms.

[4] Allaix ME, Fisichella PM, Noth I, Herbella FA, Borraez Segura B, Patti MG. (2014) Idiopathic pulmonary fibrosis and gastroesophageal reflux. implications for treatment 18(1):100-105 10.1007/s11605-013-2333-z 24002768

[5] American Thoracic Society; European Respiratory Society (2002) American Thoracic Society/ European Respiratory Society International Multidisciplinary Consensus Classification of the Idiopathic Interstitial Pneumonias. This joint statement of the American Thoracic Society (ATS) and the European Respiratory Society (ERS) was adopted by the ATS board of directors, June 2001, and by the ERS Executive Committee, June 2001. Am J Respir Crit Care Med 165(2):277–304 10.1164/ajrccm.165.2.ats01 11790668

[6] Travis WD, Costabel U, Hansell DM, King TE Jr, Lynch DA, Nicholson AG, Ryerson CJ, Ryu JH, Selman M, Wells AU, Behr J, Bouros D, Brown KK, Colby TV, Collard HR, Cordeiro CR, Cottin V, Crestani B, Drent M, Dudden RF, Egan J, Flaherty K, Hogaboam C, Inoue Y, Johkoh T, Kim DS, Kitaichi M, Loyd J, Martinez FJ, Myers J, Protzko S, Raghu G, Richeldi L, Sverzellati N, Swigris J, Valeyre D (2013) ATS/ERS committee on idiopathic interstitial pneumonias. Am J Respir Crit Care Med 188(6):733–748 10.1164/rccm.201308-1483ST PMC5803655 24032382

[7] Mueller-Mang C, Grosse C, Schmid K, Stiebellehner L, Bankier AA (2007) What every radiologist should know about Idiopathic Interstitial Pneumonias. Radiographics 27(3):595–615 10.1148/rg.273065130 17495281

[8] Piccoli M, Roccasalva F, Palmucci S, Cappello G, Mauro LA, Attinà G, Puglisi S, Vancheri C, Ettorre GC (2013) Radiological features of idiopathic interstitial pneumonia: a pictorial review. ECR Poster 2013 Vienna Austria Center doi:10.1594/ecr2013/C-2012 10.1007/s13244-014-0335-3 PMC4035488 24844883

[9] Silva CI, Müller NL, Lynch DA, Curran-Everett DA, Brown KK, Lee KS, Chung MP, Churg A (2008) Chronic hypersensitivity pneumonitis: differentiation from idiopathic pulmonary fibrosis and non-specific interstitial pneumonia by using HRCT. Radiology 246(1):288–297 10.1148/radiol.2453061881 18096541

[10] Schaefer-Prokop C, Prokop M, Fleischmann D, Herold C (2011). High-resolution CT of interstitial lung disease: key findings in common disorders. Eur Radiol.

[11] Raghu G, Collard HR, Egan JJ, Martinez FJ, Behr J, Brown KK, Colby TV, Cordier JF, Flaherty KR, Lasky JA, Lynch DA, Ryu JH, Swigris JJ, Wells AU, Ancochea J, Bouros D, Carvalho C, Costabel U, Ebina M, Hansell DM, Johkoh T, Kim DS, King TE Jr, Kondoh Y, Myers J, Müller NL, Nicholson AG, Richeldi L, Selman M, Dudden RF, Griss BS, Protzko SL, Schünemann HJ, on behalf of the ATS/ERS/JRS/ALAT Committee on Idiopathic Pulmonary Fibrosis (2011) An official ATS/ERS/JRS/ALAT statement: idiopathic pulmonary fibrosis: evidence-based guidelines for diagnosis and management. Am J Respir Crit Care Med 183(6):788–824 10.1164/rccm.2009-040GL PMC5450933 21471066

[12] Raghu G, Weycker D, Edelsberg J, Bradford WZ, Oster G (2006) Incidence and prevalence of idiopathic pulmonary fibrosis. Am J Respir Crit Care Med 174(7):810–816 10.1164/rccm.200602-163OC 16809633

[13] Thomeer M, Demedts M, Vandeurzen K, VRGT Working Group on Interstitial Lung Diseases (2001). Registration of interstitial lung diseases by 20 centres of respiratory medicine in Flanders. Acta Clin Belg.

[14] Coultas DB, Zumwalt RE, Black WC, Sobonya RE (1994) The epidemiology of interstitial lung diseases. Am J Respir Crit Care Med 150(4):967–972 10.1164/ajrccm.150.4.7921471 7921471

[15] Kolek V (1994). Epidemiology of cryptogenic fibrosing alveolitis in Moravia and Silesia. Acta Univ Palacki Olomuc Fac Med.

[16] Demedts M, Wells AU, Antò JM, Costabel U, Hubbard R, Cullinan P, Slabbynck H, Rizzato G, Poletti V, Verbeken EK, Thomeer MJ, Kokkarinen J, Dalphin JC, Taylor AN (2001). Interstitial lung diseases:an epidemiological overview. Eur Respir J Suppl.

[17] Selman M, Pardo A (2002). Idiopathic pulmonary fibrosis: an epithelial/fibroblastic cross-talk disorder. Respir Res.

[18] Vancheri C, Failla M, Crimi N, Raghu G (2010) Idiopathic pulmonary fibrosis: a disease with similarities and links to cancer biology. Eur Respir J 35:496–504 10.1183/09031936.00077309 20190329

[19] Sverzellati N (2013) Highlights of HRCT imaging in IPF. Respir Res 14(Suppl 1):S3 10.1186/1465-9921-14-S1-S3 PMC3643237 23734841

[20] Hansell DM, Bankier AA, MacMahon H, MacLoud TC, Müller NL, Remy J (2008) Fleischner Society: glossary of term for thoracic imaging. Radiology 246(3):697–722 10.1148/radiol.2462070712 18195376

[21] Katzenstein AL, Myers JL (2000). Nonspecific interstitial pneumonia and the other idiopathic interstitial pneumonias: classification and diagnostic criteria. Am J Surg Pathol.

[22] Travis WD, Matsui K, Moss J, Ferrans VJ (2000). Idiopathic nonspecific interstitial pneumonia: prognostic significance of cellular and fibrosing patterns: survival comparison with usual interstitial pneumonia and desquamative interstitial pneumonia. Am J Surg Pathol.

[23] Kim DS, Collard HR, King TE Jr (2006) Classification and natural history of idiopathic interstitial pneumonias. Proc Am Thorac Soc 3(4):285–292 10.1513/pats.200601-005TK PMC2658683 16738191

[24] du Bois R, King TE Jr (2007) Challenges in pulmonary fibrosis x 5: the NSIP/UIP debate. Thorax 62(11):1008–1012 10.1136/thx.2004.031039 PMC2117119 17965079

[25] Romagnoli M, Nannini C, Piciucchi S, Girelli F, Gurioli C, Casoni G, Ravaglia C, Tomassetti S, Gurioli C, Gavelli G, Carloni A, Dubinie A, Cantini F, Chilosi M, Poletti V (2011) Idiopathic nonspecific interstitial pneumonia: an interstitial lung disease associated with autoimmune disorders? Eur Respir J 38:384–391 10.1183/09031936.00094910 21273390

[26] Martinez FJ (2006) Idiopathic interstitial pneumonias: usual interstitial pneumonia versus nonspecific interstitial pneumonia. Proc Am Thorac Soc 3:81–95 10.1513/pats.200511-123JH 16493155

[27] Arakawa H, Yamada H, Kurihara Y, Nakajima Y, Takeda A, Fukushima Y, Fujioka M (2003). Nonspecific interstitial pneumonia associated with polymyositis and dermatomyositis: serial high-resolution CT findings and functional correlation. Chest.

[28] Screaton NJ, Hiorns MP, Lee KS, Franquet T, Johkoh T, Fujimoto K, Ichikado K, Colby TV, Müller NL (2005) Serial high resolution CT in non-specific interstitial pneumonia: prognostic value of the initial pattern. Clin Radiol 60(1):96–104 10.1016/j.crad.2004.06.029 15642299

[29] Park IN, Jegal Y, Kim DS, Do KH, Yoo B, Shim TS, Lim CM, Lee SD, Koh Y, Kim WS, Kim WD, Jang SJ, Kitaichi M, Nicholson AG, Colby TV (2009) Clinical course and lung function change of idiopathic nonspecific interstitial pneumonia. Eur Respir J 33(1):68–76 10.1183/09031936.00158507 18829672

[30] Travis WD, Hunninghake G, King TE Jr, Lynch DA, Colby TV, Galvin JR, Brown KK, Chung MP, Cordier JF, du Bois RM, Flaherty KR, Franks TJ, Hansell DM, Hartman TE, Kazerooni EA, Kim DS, Kitaichi M, Koyama T, Martinez FJ, Nagai S, Midthun DE, Müller NL, Nicholson AG, Raghu G, Selman M, Wells A (2008) Idiopathic nonspecific interstitial pneumonia: report of an American Thoracic Society project. Am J Respir Crit Care Med 177(12):1338–1347 10.1164/rccm.200611-1685OC 18388353

[31] Akira M, Inoue G, Yamamoto S, Sakatani M (2000) Non-specific interstitial pneumonia: findings on sequential CT scans of nine patients. Thorax 55:854–859 10.1136/thorax.55.10.854 PMC1745608 10992538

[32] Kim DS, Collard HR, King TE Jr (2006) Classification and natural history of the idiopathic interstitial pneumonias. Proc Am Thorac Soc 3(4):285–292 10.1513/pats.200601-005TK PMC2658683 16738191

[33] Jeong YJ, Lee KS, Müller NL, Chung MP, Chung MJ, Han J, Colby TV, Kim S (2005). Usual interstitial pneumonia and non-specific interstitial pneumonia: serial thin-section CT findings correlated with pulmonary function. Korean J Radiol.

[34] Park JS, Lee KS, Kim JS, Park CS, Suh YL, Choi DL et al (1995) Nonspecific interstitial pneumonia with fibrosis: radiographic and CT findings in seven patients. Radiology 195(3):645–648 10.1148/radiology.195.3.7753988 7753988

[35] Kim TS, Lee KS, Chung MP, Han J, Park JS, Hwang JH, Kwon OJ, Rhee CH (1998) Nonspecific interstitial pneumonia with fibrosis: high-resolution CT and pathologic findings. AJR Am J Roentgenol 171(6):1645–1650 10.2214/ajr.171.6.9843306 9843306

[36] Hartman TE, Swensen SJ, Hansell DM, Colby TV, Myers JL, Tazelaar HD, Nicholson AG, Wells AU, Ryu JH, Midthun DE, du Bois RM, Müller NL (2000) Nonspecific interstitial pneumonia: variable appearance at high-resolution chest CT. Radiology 217(3):701–705 10.1148/radiology.217.3.r00nv31701 11110931

[37] Schmidt SL, Sundaram B, Flaherty KR (2009) Diagnosing fibrotic lung disease: when is high-resolution computed tomography sufficient to make a diagnosis of idiopathic pulmonary fibrosis? Respirology 14(7):934–939 10.1111/j.1440-1843.2009.01626.x 19740255

[38] Niewoehner DE, Kleinerman J, Rice DB (1974) Pathologic changes in the peripheral airways of young cigarette smokers. N Engl J Med 291:755 10.1056/NEJM197410102911503 4414996

[39] Myers JL, Veal CF Jr, Shin MS, Katzenstein AL (1987) Respiratory bronchiolitis causing interstitial lung disease. A clinicopathologic study of six cases. Am Rev Respir Dis 135:880 10.1164/arrd.1987.135.4.880 3565934

[40] Yousem SA, Colby TV, Gaensler EA (1989). Respiratory bronchiolitis-associated interstitial lung disease and its relationship to desquamative interstitial pneumonia. Mayo Clin Proc.

[41] Fraig M, Shreesha U, Savici D, Katzenstein (2002). Respiratory bronchiolitis: a clinicopathologic study in current smokers, ex-smokers, and never-smokers. Am J Surg Pathol.

[42] Caminati A, Harari S (2006) Smoking-related interstitial pneumonias and pulmonary Langerhans cell histiocytosis. Proc Am Thorac Soc 3:299–306 10.1513/pats.200512-135TK 16738193

[43] Moon J, du Bois RM, Colby TV, Hansell DM, Nicholson AG (1999) Clinical significance of respiratory bronchiolitis on open lung biopsy and its relationship to smoking related interstitial lung disease. Thorax 54:1009–1014 10.1136/thx.54.11.1009 PMC1745385 10525560

[44] Attili AK, Kazerooni EA, Gross BH, Flaherty KR, Myers JL, Martinez FJ (2008) Smoking related interstitial lung diseases-radiologic/clinical/pathologic correlation. RadioGraphics 28:1383–1398 10.1148/rg.285075223 18794314

[45] Ryu JH, Myers JL, Capizzi SA, Douglas WW, Vassallo R, Decker PA (2005) Desquamative interstitial pneumonia and respiratory bronchiolitis-associated interstitial lung disease. Chest 127:178 10.1378/chest.127.1.178 15653981

[46] Nakanishi M, Demura Y, Mizuno S, Ameshima S, Chiba Y, Miyamori I, Itoh H, Kitaichi M, Ishizaki T (2007) Changes in HRCT findings in patients with respiratory bronchiolitis-associated interstitial lung disease after smoking cessation. Eur Respir J 29:453 10.1183/09031936.00015506 17135233

[47] Portnoy J, Veraldi KL, Schwarz MI, Schwarz MI, Cool CD, Curran-Everett D, Cherniack RM, King TE Jr, Brown KK (2007) Respiratory bronchiolitis-interstitial lung disease: long-term outcome. Chest 131:664 10.1378/chest.06-1885 17356078

[48] Aubry MC, Wright JL, Myers JL (2002). The pathology of smoking-related lung diseases. Clin Chest Med.

[49] Heynemann LE, Ward S, Lynch DA, Remy-Jardin M, Johkoh T, Müller NL (1999) Respiratory bronchiolitis, respiratory bronchiolitis-associated interstitial lung disease, and desquamative interstitial pneumonia: different entities or part of the spectrum of the same disease process? AJR Am J Roentgenol 173:1617–1622 10.2214/ajr.173.6.10584810 10584810

[50] Park JS, Brown KK, Tuder RM, Hale VA, King TE, Lynch DA (2002). Respiratory bronchiolitis-associated interstitial lung disease: radiologic features with clinical and pathologic correlation. J Comput Assist Tomogr.

[51] Liebow AA, Steer A, Billingsley JG (1965). Desquamative interstitial pneumonia. Am J Med.

[52] Carrington CB, Gaensler EA, Coutu RE, Fitzgerald MX, Gupta RG (1978). Natural history and treated course of usual and desquamative interstitial pneumonia. N Engl J Med.

[53] Swartz JS, Chatterjee S, Parambil JG (2010) Desquamative interstitial pneumonia as the initial manifestation of systemic sclerosis. J Clin Rheumatol 16:284–286 10.1097/RHU.0b013e3181eed86d 20808169

[54] Esmaeilbeigi F, Juvet S, Hwang D, Mittoo S (2012). Desquamative interstitial pneumonitis in a patient with systemic lupus erythematosus. Can Respir J.

[55] Ishii H, Iwata A, Sakamoto N, Mizunoe S, Mukae H, Kadota JI (2009). Desquamative interstitial pneumonia (DIP) in a patient with rheumatoid arthritis: is DIP associated with autoimmune disorders?. Inter Med.

[56] Radiopaedia desquamative interstitial pneumonia. http://www.radiopaedia.org/articles/desquamative-interstitial-pneumonia

[57] Hartman TE, Primack SL, Swensen SJ, Hansell D, McGuinness G, Müller NL (1993). Desquamative interstitial pneumonia: thin-section CT findings in 22 patients. Radiology.

[58] Charcot JM (1878). Des penumonies chroniques. Rev Mensuelle Med Chir.

[59] Milne LS (1911). Chronic pneumonia (including a discussion of two cases of syphilis of the lung). Am J Med Sci.

[60] Davison AG, Heard BE, McAllister WAC, Turner-Warwick ME (1983). Cryptogenic organizing pneumonitis. Q J Med.

[61] Lee JW, Lee KS, Lee HY, Chung MP, Yi CA, Kim TS, Chung MJ (2010) Cryptogenic organizing pneumonia: serial high-resolution CT findings in 22 patients. Am J Roengtenol 195:916–922 10.2214/AJR.09.3940 20858818

[62] Cordier JF (2006) Cryptogenic organising pneumonia. Eur Respir J 28(2):422–446 10.1183/09031936.06.00013505 16880372

[63] Epperly MW, Guo H, Gretton JE, Greenberger JS (2003) Bone marrow origin of myofibroblasts in irradiation pulmonary fibrosis. Am J Respir Cell Mol Biol 29(2):213–224 10.1165/rcmb.2002-0069OC 12649121

[64] Lazor R, Vandevenne A, Pelletier A, Leclerc P, Court-Fortune I, Cordier JF (2000) Cryptogenic organizing pneumonia. Characteristics of relapses in a series of 48 patients. The Groupe d’Etudes et de Reserche sur les Maladles “Orphelines” Pulmonaires (GERM”O”P). Am J Respir Crit Care Med 162(2 Pt 1):571–577 10.1164/ajrccm.162.2.9909015 10934089

[65] Roberton BJ, Hansell DM (2011) Organizing pneumonia: a kaleidoscope of concepts and morphologies. Eur Radiol 21(11):2244–2254 10.1007/s00330-011-2191-6 21744289

[66] Marlow TJ, Krapiva TI, Scahbel SI, Judson MA (1999) The “fairy ring”: a new radiographic finding in sarcoidosis. Chest 115(1):275–276 10.1378/chest.115.1.275 9925098

[67] Agarwai R, Aggarwai AN, Gupta D (2007) Another cause of reverse halo sign: Wegener’s granulomatosis. Br J Radiol 80(958):849–850 10.1259/bjr/61353689 17959924

[68] Lee KS, Kullnig P, Hartman TE, Muller NL (1994) Cryptogenic organizing pneumonia: CT findings in 43 patients. AJR Am J Roentgenol 162(3):543–546 10.2214/ajr.162.3.8109493 8109493

[69] Muller NL, Staples CA, Miller RR (1990) Bronchiolitis obliterans organizing pneumonia: CT features in14 patients. AJR Am J Roentgenol 154:983–987 10.2214/ajr.154.5.2108572 2108572

[70] Vourlekis JS, Brown KK, Cool CD, Young DA, Cherniack RM, King TE, Schwarz MI (2000). Acute interstitial pneumonitis. Case series and review of the literature. Medicine (Baltimore).

[71] Hamman L, Rich A (1944). Acute diffuse interstitial fibrosis of the lung. Bulls Johns Hopkins Hosp.

[72] Ichikado K, Johkoh T, Ikezoe J, Takeuchi N, Kohno N, Arisawa J, Nakamura H, Nagareda T, Itoh H, Ando M (1997) Acute interstitial pneumonia: high-resolution CT findings correlated with pathology. AJR Am J Roentgenol 168(2):333–338 10.2214/ajr.168.2.9016201 9016201

[73] Katzenstein AL, Mukhopadhyay S, Zanardi C, Dexter E (2010) Clinically occult interstitial fibrosis in smokers: classification and significance of a surprisingly common finding in lobectomy specimens. Hum Pathol 41:316 10.1016/j.humpath.2009.09.003 20004953

[74] Ferguson EC, Berkowitz EA (2012) Lung CT: Part 2, The Interstitial Pneumonias—Clinical, Histologic, and CT Manifestations. Am J Roentgenol 199:W464–W476 10.2214/AJR.10.7309 22997396

[75] Mukhopadhyay S, Parambil JG (2012) Acute interstitial pneumonia (AIP): relationship to Hamman-Rich syndrome, diffuse alveolar damage (DAD), and acute respiratory distress syndrome (ARDS). Semin Respir Crit Care Med 33:476–485 10.1055/s-0032-1325158 23001802

[76] Bernard GR, Artigas A, Brigham KL, Carlet J, Falke K, Hudson L, Lamy M, Legall JR, Morris A, Spragg R (1994) The American-European Consensus Conference on ARDS. Definitions, mechanisms, relevant outcomes, and clinical trial coordination. Am J Respir Critic Care Med 149(3 Pt 1):818–824 10.1164/ajrccm.149.3.7509706 7509706

[77] Ichikado K, Suga M, Müller NL, Taniguchi H, Kondoh Y, Akira M, Johkoh T, Mihara N, Nakamura H, Takahashi M, Ando M (2002) Acute interstitial pneumonia: comparison of high-resolution computed tomography findings between survivors and nonsurvivors. Am J Respir Crit Care Med 165(11):1551–1556 10.1164/rccm.2106157 12045132

[78] Savici D, Katzenstein AL (2001) Diffuse alveolar damage and recurrent respiratory failure: report of 6 cases. Human Pathol 32:1398–1402 10.1053/hupa.2001.29670 11774176

[79] Akira M, Hamada H, Sakatani M, Kobayashi C, Nishioka M, Yamamoto S (1997) CT findings during phase of accelerated deterioration in idiopathic pulmonary fibrosis. AJR Am J Roengtenol 168(1) 10.2214/ajr.168.1.8976924 8976924

[80] Primack SL, Hartman TE, Ikezoe J, Akira M, Sakatani M, Müller NL (1993) Acute interstitial pneumonia: radiographic and CT findings in nine patients. Radiology 188(3):817–820 10.1148/radiology.188.3.8351354 8351354

[81] Johkoh T, Müller NL, Taniguchi H, Kondoh Y, Akira M, Ichikado K, Ando M, Honda O, Tomiyama N, Nakamura H (1999) Acute interstitial pneumonia: thin-section CT findings in 36 patients. Radiology 211:859–863 10.1148/radiology.211.3.r99jn04859 10352616

[82] Wittram C, Mark EJ, McLoud TC (2003) CT-histologic correlation of the ATS/ERS 2002 classification of idiopathic interstitial pneumonias. Radiographics 23(5):1057–1071 10.1148/rg.235035702 12975500

[83] Johkoh T, Müller NL, Cartier Y, Kavanagh PV, Hartman TE, Akira M, Ichikado K, Ando M, Nakamura H (1999) Idiopathic interstitial pneumonias: diagnostic accuracy of thin-section CT in 129 patients. Radiology 211(2):555–560 10.1148/radiology.211.2.r99ma01555 10228542

[84] Desai SR, Wells AU, Rubens MB, Evans TW, Hansell DM (1999) Acute respiratory distress syndrome: CT abnormalities at long-term follow-up. Radiology 210:29–35 10.1148/radiology.210.1.r99ja2629 9885583

[85] Kobayashi H, Itoh T, Sasaki Y, Konishi J (1996). Diagnostic imaging of idiopathic adult respiratory distress syndrome (ARDS)/diffuse alveolar damage (DAD): histologic correlation with radiological imaging. Clin Imaging.

[86] Liebow A, Carrington CB, Simon M, Potchen EJ, LeMay M (1969). The interstitial pneumonias. Frontiers of pulmonary radiology.

[87] Swigris JJ, Berry GJ, Raffin TA, Kuschner WG (2002). Lymphoid interstitial pneumonia: a narrative review. Chest.

[88] Liebow A, Carrington C (1973). Diffuse pulmonary lymphoreticular infiltrations associated with dysproteinemia. Med Clin North Am.

[89] Cha SI, Fessler MB, Cool CD, Schwarz MI, Brown KK (2006) Lymphoid interstitial pneumonia: clinical features, associations and prognosis. Eur Respir J 28(2):364–369 10.1183/09031936.06.00076705 16571614

[90] Koss M, Hochholzer L, Langloss J, Wehunt WD, Lazarus AA (1987). Lymphoid interstitial pneumonia: clinicopathological and immunopathologic findings in 18 cases. Pathology.

[91] Koss MN (1995). Pulmonary lymphoid disorders. Semin Diagn Pathol.

[92] Ichikawa Y, Kinoshita M, Koga T, Oizumi K, Fujimoto K, Hayabuchi N (1994). Lung cyst formation in lymphocytic interstitial pneumonia: CT features. J Comput Assist Tomogr.

[93] Silva CI, Flint JD, Levy RD, Müller NL (2006) Diffuse lung cysts in lymphoid interstitial pneumonia: high-resolution CT and pathologic findings. J Thorac Imaging 21(3):241–244 10.1097/01.rti.0000213554.61752.73 16915074

[94] Johkoh T, Ichikado K, Akira M, Honda O, Tomiyama N, Mihara N, Kozuka T, Koyama M, Hamada S, Nakamura H (2000). Lymphocytic interstitial pneumonia: follow-up CT findings in 14 patients. J Thorac Imaging.

[95] Lee KH, Lee JS, Lynch DA, Song KS, Lim TH (2002). The radiologic differential diagnosis of diffuse lung diseases characterized by multiple cysts or cavities. J Comput Assist Tomogr.

[96] Hare SS, Souza CA, Bain G, Seely JM, Gomes MM, Quigley M (2012) The radiological spectrum of pulmonary lymphoproliferative disease. Br J Radiol 85:848–864 10.1259/bjr/16420165 PMC3474050 22745203

[97] Gotway MB, Freemer MM, King TE Jr (2007) Challenges in pulmonary fibrosis. 1: Use of high resolution CT scanning of the lung for the evaluation of patients with idiopathic interstitial pneumonias. Thorax 62(6):546–553 10.1136/thx.2004.040022 PMC2117220 17536033

[98] Honda O, Johkoh T, Ichikado K, Tomiyama N, Maeda M, Mihara N, Higashi M, Hamada S, Naito H, Yamamoto S, Nakamura H (1999) Differential diagnosis of lymphocytic interstitial pneumonia and malignant lymphoma on high-resolution CT. AJR Am J Roentgenol 173(1):71–74 10.2214/ajr.173.1.10397102 10397102

[99] Amitani R, Niimi A, Kuse F (1992). Idiopathic pulmonary upper lobe fibrosis (IPUF). Kokyu.

[100] Reddy TL, Tominaga M, Hansell DM, von der Thusen J, Rassl D, Parfrey H, Guy S, Twentyman O, Rice A, Maher TM, Renzoni EA, Wells AU, Nicholson AG (2012) Pleuroparenchymal fibroelastosis: a spectrum of histopathological and imaging phenotypes. Eur Respir J 40(2):377–385 10.1183/09031936.00165111 22441748

[101] Fujikura Y, Kanoh S, Kouzaki Y, Hara Y, Matsubara O, Kawana A (2014). Pleuroparenchymal fibroelastosis as a series of airway complications associated with chronic graft-versus-host disease following allogeneic bone marrow transplantation. Intern Med.

[102] Kusagaya H, Nakamura Y, Kono M, Kaida Y, Kuroishi S, Enomoto N, Fujisawa T, Koshimizu N, Yokomura K, Inui N, Suda T, Colby TV, Chida K (2012) Idiopathic pleuroparenchymal fibroelastosis: consideration of a clinicopathological entity in a series of Japanese patients. BMC Pulm Med 12:72 10.1186/1471-2466-12-72 PMC3539991 23216996

[103] Koulaouzidis A, Karagiannidis A, Prados S, Pattenshetty D, Deramon A, Tan WC (2006). Lymphocytic interstitial pneumonitis (LIP)—the liver and the lung. Ann Hepatol.

